# The exosomal miR-26b-3p derived from Crohn’s disease-associated mesenteric adipose tissue induces M1 macrophage polarization and exacerbates ileocolonic anastomosis inflammation via the p38-MAPK signaling pathway

**DOI:** 10.3389/fimmu.2026.1754302

**Published:** 2026-02-25

**Authors:** Enhao Wu, Wenwei Qian, Xi Zhang, Lili Gu, Zhen Guo, Zeqian Yu, Yi Li, Weiming Zhu

**Affiliations:** 1 Department of General Surgery, Jinling Hospital, Affiliated Hospital of Medical School, Nanjing University, Nanjing, Jiangsu, China; 2 Department of General Surgery, The Fourth Affiliated Hospital of Soochow University, Soochow University, Suzhou, Jiangsu, China; 3 Department of General Surgery, Jinling Hospital, Medical School of Southeast University, Nanjing, Jiangsu, China; 4 Department of General Surgery, Nanjing Women and Children’s Healthcare Hospital, Women’s Hospital of Nanjing Medical University, Nanjing Medical University, Nanjing, Jiangsu, China

**Keywords:** Crohn’s disease, exosome, macrophage M1 polarization, mesenteric adipose tissue, miR-26b-3p

## Abstract

**Purpose:**

Crohn’s Disease (CD) is a chronic inflammatory condition characterized by intestinal inflammation, especially in the progression of postoperative anastomotic recurrence. Recent evidence implicates mesenteric adipose tissue (MAT) in CD pathogenesis, particularly through its exosome secretion, which may influence inflammation pathways. The molecular mechanisms driving this inflammation remain inadequately understood.

**Methods:**

Exosomes were isolated from MAT of the diseased bowel and macroscopically normal MAT from the surgical margins of patients with CD. We induced chronic intestinal inflammation in mice using dinitrobenzene sulfonic acid (DNBS), simulating CD-like MAT. Using a surgical model of IL10-knockout mice, we performed a series of experiments *in vitro* and *in vivo* to assess the effects of exosomes on ileocolonic anastomosis inflammation and macrophage M1 polarization. We performed microRNA microarray analysis, colonoscopy, Western blotting, luciferase assays, and immunofluorescence to investigate the underlying mechanisms.

**Results:**

Hypertrophic MAT-Exosomes (Ht-exos) promoted ileocolonic anastomotic inflammation by activating macrophage M1 polarization in CD. *In vivo*, injection of Ht-exos induced inflammatory tissue damage and macrophage M1 polarization in an IL-10^-/-^ mouse model of ileocecal resection. *In vitro*, Ht-exos was found to promote macrophage inflammatory response and M1 polarization through the activation of the p38-MAPK pathway. Further, exosomal miR-26b-3p was enriched in MAT-Exosomes and involved in exosome-mediated inflammation activation. Mechanistically, hypertrophic MAT released exosomal miR-26b-3p and promoted inflammation by targeting tripartite motif-containing 33 (TRIM33) via the p38-MAPK signaling pathway and promoting macrophage M1 polarization. Furthermore, miR-26b-3p expression was positively correlated with the degree of ileocolonic anastomosis inflammation in CD.

**Conclusion:**

Our findings reveal that exosomal miR-26b-3p drives widespread macrophage inflammation and M1 polarization in hypertrophic MAT-induced ileocolonic anastomosis inflammation via the MAPK pathway.

## Introduction

Crohn’s disease (CD) is a chronic inflammatory disorder of the gastrointestinal tract, with an unknown etiology. It is characterized by transmural inflammation and clinical symptoms such as abdominal pain, diarrhea, and weight loss (1). Notably, approximately 70% of patients with CD require surgical intervention, with a significant proportion of them experiencing postoperative anastomotic inflammation that often requires repeat surgeries (2). Therefore, there is an urgent need to reduce anastomotic recurrence.

Among the hallmark features of CD is “creeping fat” and hypertrophy of mesenteric adipose tissue (HtMAT), which is closely associated with disease severity (3, 4). The relationship between MAT and anastomotic inflammation remains a key focus in surgical management (5, 6). Targeting MAT inflammation may alleviate disease progression (7, 8). Accordingly, elucidating the influence of MAT on anastomotic healing is important to inform clinical decision-making.

Macrophages play a crucial role in CD pathogenesis by sensing microbial-associated molecular patterns through innate immune receptors, including toll-like receptors and nucleotide-binding oligomerization domain-like receptors (9, 10). Subsequently, the macrophages produce pro-inflammatory cytokines, including interleukin 1-beta (IL-1β), IL-6, and tumor necrosis factor-alpha (TNF-α), which drive mucosal inflammation and immune cell activation, especially in the early stages of CD (11, 12). Therefore, macrophages have emerged as promising therapeutic targets and key regulators of intestinal immune homeostasis. Therefore, elucidating their roles in disease progression is crucial.

Exosomes, critical mediators of intercellular communication, have gained increased attention recently (13). MAT influences surrounding tissues and distant organs by secreting various factors, including exosomes (14), which can modulate processes in the heart, brain, and liver (15–17). Gut microbiota and preadipocyte-derived exosomes can affect intestinal fibrosis (18, 19), which suggests the potential role of exosomes from MAT in intestinal inflammation. Exosomes derived from adipose tissue can influence the direction of macrophage polarization (20–22). However, their specific contributions to ileocolonic anastomotic inflammation in CD remain unclear.

The impact of Ileocolonic anastomosis inflammation, which is influenced by HtMAT in CD, remains unclear. However, given the important role of Exosomes in intercellular interaction between macrophages and MAT, we speculated that HtMAT regulate the phenotype and function of decidual macrophages by secreting EXOs-miRNA, consequently promoting the ileocolonic anastomosis inflammation in CD. Therefore, this study aimed to investigate the role of MAT in anastomotic inflammation by using an ileocecal anastomosis mouse model.

## Material and methods

### MAT from patients with CD

Mature adipocytes were isolated as previously described, with minor modifications (23, 24). Hypertrophic MAT (HtMAT) from the diseased bowel and macroscopically normal MAT (nMAT) were intraoperatively obtained from 15 patients with CD (Jinling Hospital, 2023–2024; patient details shown in **Supplementary Tables 1**, **2**. The hypertrophic MAT samples and the control adipose tissue samples were obtained from the same patient during surgery. The collected tissues were immediately transported on ice and processed within 20 min. After removal of blood using Hank’s balanced salt solution buffer, vascular components were excised, followed by mincing of tissues prior to enzymatic digestion (3% bovine serum albumin [BSA], 8 mg/mL collagenase D, 2.4 U/mL Dispase II; 37 °C, 1 h). Finally, digested samples were filtered (200 μm), and adipocytes were collected through centrifugation (300 × *g*, 5 min, 4 °C).

### Cell lines

All cell lines were maintained at 37 °C using 5% CO_2_ with corresponding medium. Tohoku Hospital Pediatrics-1 (THP-1, CVCL_0006), Immortalized bone marrow-derived macrophages (iBMDMs) and 3T3-L1 cells (BFN608006390) were obtained from the National Collection of Authenticated Cell Cultures (Shanghai, China). 3T3-L1 pre-adipocytes were maintained in Dulbecco’s modified Eagle medium (DMEM) supplemented with 10% fetal bovine serum (FBS) (Gibco, Gaithersburg, MD, USA) and penicillin/streptomycin. At 2 days post confluence (designated as Day 0), a differentiation cocktail (10μg/mL insulin, dexamethasone, and 0.5 mM 3-isobutyl-1-methyl-xanthine) was added to the cells in fresh media. After 48 h, the media was changed to DMEM supplemented with 10% FBS containing 10μg/mL insulin, which was replaced every alternate day. Eight days after induction (designated Day 8), cells were harvested for subsequent analyses.

### Isolation and identification of exosomes

Exosomes were isolated and purified as previously described (19). For exosome isolation, adipose tissue samples were precisely weighed to ensure equal starting mass (100 mg wet weight per sample) before homogenization. Adipocytes were cultured in exosome-free medium for 48 h. Next, the supernatant was collected and sequentially centrifuged at 4 °C (300 × *g*, 10 min; 3,000 × *g*, 20 min) to remove cells and debris. Exosomes were isolated via ultracentrifugation (10,000 × *g*, 30 min; 100,000 × *g*, 70 min), followed by an additional 100,000 × *g* wash (70 min). Purified exosomes were stored at -80 °C. Exosome morphology and particle size distribution were assessed using transmission electron microscopy (TEM; FEI Tecnai Spirit, 120 kV) and nanoparticle tracking analysis (NTA; Malvern LM10), respectively. Protein content was quantified via the bicinchoninic acid assay (Thermo Fisher Scientific) after exosome lysis in radioimmunoprecipitation assay (RIPA) buffer. Exosome identity was confirmed using western blotting for surface markers (TSG101, CD9, and CD63).

### qRT-PCR

Total RNA was isolated from cells and exosomes using TRIzol Reagent (Invitrogen), with its concentration and purity being assessed using Nanodrop 2000 (Thermo Fisher Scientific). cDNA was synthesized using SuperScript III Reverse Transcriptase (Invitrogen) for mRNA or Bulge-Loop microRNA (miRNA) qRT-PCR Starter Kit (RiboBio) for miRNAs. Moreover, qPCR was performed on an ABI 7300 system (Applied Biosystems) using SYBR Premix Ex Taq (Takara Bio, Shiga, Japan). **Supplementary Table 3** presents the primer sequences for the target genes (TNF-α, IL-1β, IL-6, iNOS, CD86, CD206, Arg1) and controls (β-actin). Gene expression was normalized to β-actin (mRNA) and each sample was run in triplicate, and all reactions were repeated three times to ensure reproducibility of the results.

### Western blot analysis

Total protein was extracted from cells and exosomes using RIPA lysis buffer (KeyGen Biotechnology), followed by assessment of protein content using the bicinchoninic acid assay (Thermo Fisher Scientific). Proteins (10–30 μg per sample) were separated using 10% sodium dodecyl sulfate-polyacrylamide gel electrophoresis (Wanlei) and transferred to polyvinylidene difluoride membranes (Millipore). After blocking with 5% non-fat milk (1 h, room temperature), the membranes were incubated with primary antibodies overnight at 4 °C, followed by species-matched secondary antibodies conjugated with horseradish peroxidase (1 h, room temperature). Protein bands were visualized using ECL reagent (Zen-Bio) and imaged using a Tanon 5200 chemiluminescence system (Tanon Science & Technology).

### Flow cytometric analysis

iBMDMs were incubated with 30 μg/ml exosomes for 48 h. For immunophenotyping, the cells were harvested and blocked with 3% BSA in PBS for 30 min, followed by incubation with phycoerythrin-conjugated CD11b and allophycocyanin-conjugated CD86 (BioLegend; San Diego, CA), with appropriate isotype controls in the dark cells. In flow cytometry analysis, gating was sequentially applied as follows: cells were first selected based on forward- and side-scatter properties (FSC for size, SSC for granularity) to exclude debris; then, doublets were excluded using FSC-A versus FSC-H; finally, live cells were identified by DAPI negativity. For surface marker analysis (CD11b, CD86), fluorescence thresholds were set using fluorescence-minus-one (FMO) controls. Samples were analyzed using a FACSCalibur flow cytometer (BD Biosciences), and data were processed using FlowJo software (v10.0).

### Luciferase reporter assay

The 3’-UTR of suppressor of tripartite motif-containing 33 (TRIM33) (wild-type [WT] or mutant [MUT] containing the miR-26b-3p binding site) was synthesized using GenScript and cloned into the pGL3-REPORT vector (FseI/XbaI sites). Macrophages were transfected with miR-mimic or miR-NC, plated in 96-well plates, and subsequently co-transfected with either the pGL3-TRIM33-WT or pGL3-TRIM33-MUT construct. Firefly and Renilla luciferase activities were measured at 48 h after transfection using the Dual-Luciferase Reporter Assay System (Promega), with Renilla luciferase as the internal control.

### Exosomal miRNA sequencing

Total RNA was pooled from three pairs of Crohn-exos and Control-exos and subjected to miRNA sequencing at Lianchuan Biotech. Sequencing libraries of small RNAs (length: 18–30 nucleotides) were generated following the manufacturer’s instructions. Library sequencing was performed using the Illumina HiSeq 2500 platform, followed by annotation of small RNAs using miRBase v22. Differential expression analyses were performed based on the read numbers and pairwise comparisons. Differentially expressed miRNAs were identified using fold-change and p-value filtering. Total RNA was isolated using TRIzol reagent, followed by quantification and quality assessment using NanoDrop 2000 (Thermo Fisher). RNA integrity and gDNA contamination tests were performed using denaturing agarose gel electrophoresis. Before RNA-seq library construction, ribosomal RNA was removed using the RiboMinus Eukaryote Kit (Qiagen). The sequencing library was established using an Agilent 2100 Bioanalyzer with an Agilent DNA 1000 Chip Kit (Agilent, CA, USA), with adjustment to 10 nM prior to cluster generation. cDNA was sequenced using a HiSeq 2000 system (Illumina, San Diego, CA, USA) and a 100-bp paired-end run.

### Exosome uptake by macrophages and tracking *ex vivo*

Exosomes were fluorescently labeled using DiI (Sigma-Aldrich) or DiR (Invitrogen) following the manufacturer’s guidelines. Briefly, exosomes were incubated with the dye (2 mg/mL, 10 min), followed by ultracentrifugation (100,000 × *g*, 1 h) to remove unbound dye. Next, macrophages were treated with Dil-labeled exosomes (10 μg/mL, 12 h) and washed with phosphate buffered saline to remove non-internalized exosomes, followed by counterstaining of nuclei using DAPI (Thermo Fisher). Internalization was visualized using a confocal microscope (Zeiss LSM880). Subsequently, intraperitoneal (IP) injections of DiR-labeled exosomes (200 μg) were administered to mice. After 12 h, the fluorescence distribution in anastomotic tissues was assessed using an IVIS Lumina II imaging system (PerkinElmer).

### Histological evaluation

Anastomosis and colon samples were fixed in 4% formaldehyde for 48 h. Next, they were gradually dehydrated, embedded in paraffin, cut into 4-μm sections, and stained with hematoxylin and eosin. Stained sections were observed through light microscopy, followed by calculation of the severity of histological inflammation as previously reported. Five aspects were considered: mucosal architecture, infiltration of mononuclear cells and neutrophils, epithelial defects, and goblet cell loss (**Supplementary Table 4**).

### Cell transfections and transductions

The miR-26b-3p mimic (miR-mimic) and negative control (miR-NC) were obtained from RiboBio and transfected into cells at 50% confluence using Lipofectamine 2000 (Invitrogen) following the manufacturer’s instructions. Next, miR-26b-3p inhibitor was added at a final concentration of 100 nM. For mitogen-activated protein kinase (MAPK) knockdown, we used a lentiviral shRNA construct (GenePharma), with a scrambled lentiviral vector serving as a negative control. Fibroblasts at approximately 50% confluency were transduced at a multiplicity of infection of 80. Transfection and transduction efficiencies were verified using quantitative reverse transcription PCR (qRT-PCR).

### *In vitro* detection of miR-26b-3p transfer

Cy3-labeled miR-26b-3p mimics (GenePharma) were transfected into 3T3-L1 mature adipocytes following the manufacturer’s instructions. Subsequently, exosomes were isolated from the conditioned medium and co-cultured with recipient fibroblasts. After incubation, fibroblasts were fixed with 4% paraformaldehyde, permeabilized with 0.05% Triton X-100, and counterstained with DAPI. To visualize miR-26b-3p transfer, fluorescent images were captured using a Zeiss LSM880 confocal microscope.

### Clinical samples

We prospectively enrolled 13 consecutive patients with CD who underwent bowel resection for ileocolonic anastomotic strictures and 10 patients with colon cancer who underwent right hemicolectomy at Jinling Hospital in 2024. **Supplementary Table 5** shows the clinical characteristics of the included patients. We collected intestinal tissue from diseased segments or resection margins as well as the corresponding MAT from each patient. All specimens were immediately processed by dividing them into two aliquots: one snap-frozen in liquid nitrogen (-80 °C storage) and the other fixed in 10% phosphate-buffered formalin for paraffin embedding. For fibrosis assessment, Masson’s trichrome-stained sections were digitally scanned using a Nanozoomer system (Hamamatsu). The study protocol was approved by the Jinling Hospital Ethics Committee, and all participants provided written informed consent.

### Overexpression through plasmid transfection *in vitro*

TRIM33 and empty vector plasmids were purchased from GenePharma (Shanghai, China). Macrophages were seeded in six-well plates (2×105 cells/well) and incubated for 24 h to reach 60–70% confluence. Plasmid transient transfection was performed using Lipofectamine™ LTX (15338100, Thermo) following the manufacturer’s instructions. Briefly, 2.5 μg of plasmid DNA and 6.25 μL of transfection reagent were mixed and added to the cell medium without antibiotics. After 24-h incubation, cell medium was replaced with fresh complete culture medium to maintain cell growth for another 24 h before harvesting.

### miRNA target prediction

Target genes of the miRNAs were predicted using the following bioinformatics databases: TargetScan [http://www.targetscan.org ], miRDB [http://mirdb.org/], mirDIP [http://ophid.utoronto.ca/mirDIP], DIANA [https://diana.e-ce.uth.gr], and PicTar [https://pictar.mdc-berlin.de]. Additionally, we analyzed genes from three databases.

### Endoscopy

For endoscopic evaluation, mice were anesthetized using 2–3% isoflurane/O^2^, followed by extensive fecal removal using flexible feeding tubes. An Olympus URF type V endoscope was rectally inserted to a depth of ≤5 cm; moreover, videos of the endoscopic procedure were recorded using a Medicap USB200 Medical Digital Video Recorder while retracting the endoscope. Data analyses were performed as previously described (25).

### Animal models of Crohn’s disease in mice

Male C57BL/6 mice, aged 8 weeks, were obtained from the Model Animal Research Center of Nanjing University (Nanjing, China), for establishing a intestinal inflammation model. The experiment divided the mice into two groups: a model group (mHt-exos) and a control group (mN-exos), each comprising five mice. intestinal inflammation was induced using an established DNBS rectal administration protocol over six weeks (26), the mice were euthanized, and their Mesenteric Adipose Tissues were harvested.

### Animals and ileocolonic resection in IL-10^-/-^ mice

We obtained 8–10 weeks-old WT and IL-10-deficient (IL-10^-/-^) male mice from the Model Animal Research Center of Nanjing University (Nanjing, China). They underwent ileocolonic resection as previously described (27, 28). Briefly, mice were placed in a clean cage without any solid food for 24 h before the procedure. The animal was induced anesthesia by transferring the animal to the anesthesia induction chamber (3–4% isoflurane and oxygen flow of 1–2 liters/min), once the animal loses consciousness, removed the animal from the induction chamber and depilate the abdomen from sternum, placed the animal on the heating pad in dorsal recumbency with the tail toward the surgeon while the nozzle of the anesthesia system over the animal’s snout, and set it to 2–3% isoflurane and oxygen flow of 1–2 liters/min. The skin was disinfected by scrubbing with an aqueous iodophor solution, followed by 70% (vol/vol) isopropanol, then, covered the entire animal with a sterile surgical drape and created an opening in the drape to expose the surgical field. Subsequently, surgeries were performed under sterile conditions using an operating microscope (7× magnification). After ligating the mesentery, we removed the small intestine proximal to the ileocecal junction, 5 cm from the proximal colon (length, 2 cm). We used 9–0 monofilament sutures to reconstruct the continuity of the intestinal tract using an end-to-end, interrupted, single-layered anastomosis procedure. Immediately after anastomosis, saline in the WT and IL-10^-/-^ control (IL-10+saline) groups, 200 μg mN-exos in the IL-10^-/-^ group (IL-10+mN-exos), and 200 μg Ht-exos in the IL-10^-/-^ group (IL-10+mHt-exos) were directly instilled onto the external anastomotic surface in a single dose, followed by intraperitoneal (IP) injections at 4-day intervals. All mice were euthanized after 28 days. The postoperative survival rate of IL-10^-/-^mice was 80%. Additionally, we obtained a 1-cm segment of the small intestine (Anastomosis SI) and colon (Anastomosis C) proximal to the anastomosis for further analysis.

### Immunofluorescence analysis

For subsequent experiments, paraffin-embedded samples were cut into 5-μm-thick sections. Deparaffinization, rehydration, and antigen retrieval were sequentially performed. Tissue sections were blocked using 5% BSA and incubated overnight with the following primary antibodies: anti-CD86, anti-CD68, anti-F4/80, and anti-iNOS. Subsequently, they were incubated with Cy5- and FITC-conjugated secondary antibodies for 1 h at room temperature. For immunofluorescence staining, fibroblasts were fixed with 4% paraformaldehyde and permeabilized with 0.1% Triton X-100. After blocking with 5% BSA for 1 h, cells were incubated with the primary antibodies overnight at 4 °C. Next, they were incubated with FITC-conjugated secondary antibodies for 1 h at room temperature. Finally, tissue and cell slides were incubated with 0.1% DAPI solution for 10 min at room temperature. A confocal laser scanning microscope (Zeiss LSM880) was used to capture images.

### Fluorescence *in situ* hybridization

The Cy3-labeled probe for miR-26b-3p (sequence: 5'-CCUGUUCUCCAUUACUUGGCUC-3') was designed and synthesized by GenePharma. Briefly, tissues were permeabilized in 0.5% Triton X-100 for 30 min after deparaffinization. Subsequently, the slides were incubated in pre-hybridization buffer at 37 °C for 30 min. Next, Cy3-labeled miR-26b-3p probes were added and incubated with the slides in hybridization solution at 37 °C overnight. The next day, nuclei were stained with DAPI, following by examination of the slides using a confocal microscope (Zeiss LSM880).

### Statistical analysis

All statistical analyses were performed using GraphPad Prism software version 7.0 and SPSS version 20.0. Between-group comparisons of normally and non-normally distributed data were performed using Student’s t test and the Mann–Whitney U test, respectively. Among-group comparisons were performed using one-way analysis of variance with Tukey’s multiple comparison test. Correlations among the variables were assessed using Pearson’s correlation test. Statistical significance was set at *P < 0.05; **P < 0.01; ***P < 0.001; ****P < 0.0001.

## Results

### Evaluating the effect of MAT-derived exosomes from Crohn’s disease patients on macrophage M1 polarization *in vivo*

**Figure 1:**
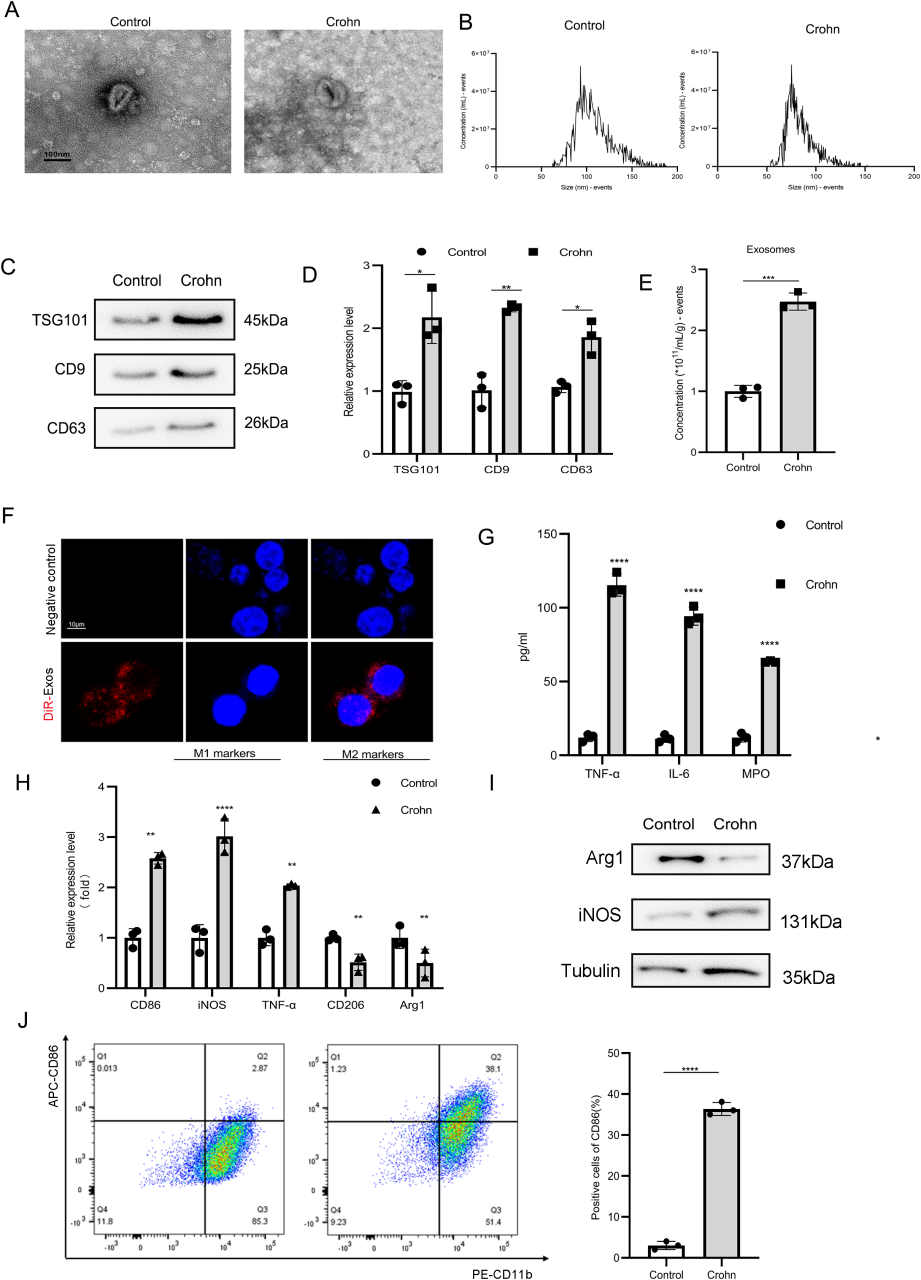
Evaluating MAT-derived exosomes from Crohn’s disease patients on macrophage M1 polarization. **(A)** Both electron micrographs of exosomes from mesenteric adipose tissue in CD patients. **(B)** Nanoparticle tracking analysis was used to detect the size of exosomes. **(C)** Exosomal protein levels of TSG101, CD9 and CD63 were detected by western blot. **(D)** qPCR validation of exosomal miRNAs levels. **(E)** The concentration of exosomes was detected by the BCA assay. **(F)** Dil-labeled exosomes were used to coculture with TPH-1s to visualize the internalization MAT-Exos by macrophage. **(G)** The concentrations of pro-inflammatory and anti-inflammatory cytokines in culture supernatants of TPH-1s. The mRNA expression levels **(H)**, the protein expression levels **(I)** and flow cytometry **(J)** were detected in TPH-1s in different groups. BCA: bicinchoninic acid. Data are expressed as means ± SD. *P < 0.05; **P < 0.01; ***P < 0.001; ****P < 0.0001 and n = 3 biological replicates.

Exosomes were isolated from HtMAT of the diseased bowel and macroscopically normal MAT from the surgical margins of patients with CD using established protocols, termed as Crohn groups and Control groups, respectively. Comparative analyses using TEM imaging showed similar cup-shaped morphology (**Figure 1A**), while NTA demonstrated comparable size distributions (30–200 nm) between both exosome populations (**Figure 1B**). Western blot analysis revealed significantly elevated levels of exosomal markers (CD9, CD63, and TSG101) in Ht-exos (**Figures 1C, D**). Moreover, an increased exosomal protein yield was observed when adipocytes were normalized (**Figure 1E**), indicating enhanced exosome biogenesis in HtMAT. THP-1s co-cultured with exosomes. Confocal microscopy confirmed effective internalization of Dil-labeled exosomes (30 μg/mL) by THP-1s within 12 h (**Figure 1F**). Crohn-exos treatment significantly altered the cytokine profile, increasing pro-inflammatory mediators, increasing pro-inflammatory mediators (TNF-α, IL-6, MPO) in culture supernatants (**Figure 1G**). At the transcriptional level, Crohn-exos upregulated M1-associated genes (CD86, iNOS and TNF-α) and downregulated M2 markers (Arg1 and CD206) (**Figure 1H**). Consistent with these findings, western blotting showed elevated M1 protein expression (**Figure 1I**), while flow cytometry confirmed an increase in CD86^+^ M1 macrophages (**Figure 1J**). Taken together, these findings demonstrate that the Exosomes divided from HtMAT of the diseased bowel in CD preferentially drive macrophage polarization toward a pro-inflammatory M1 phenotype, which mechanistically explains their role in exacerbating inflammation.

### Evaluating the effects of MAT-derived exosomes from a DNBS-induced mouse model of Crohn’s disease on macrophage M1 polarization *in vivo*

**Figure 2:**
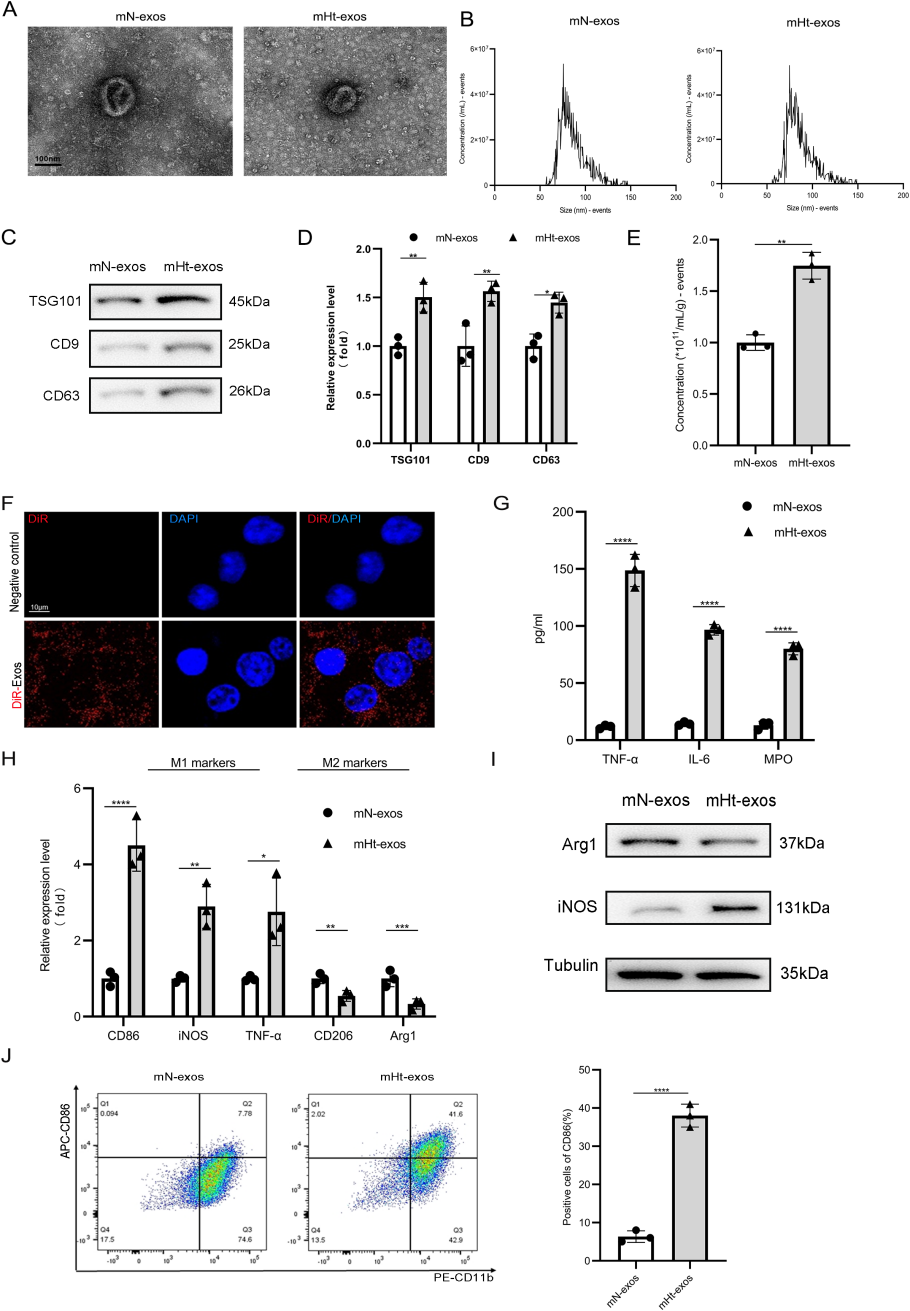
Evaluating MAT-derived exosomes from a DNBS-induced mouse on macrophage M1 polarization. **(A)** Both electron micrographs of exosomes from mesenteric adipose tissue on a DNBS-induced mouse model of CD and control groups. **(B)** Nanoparticle tracking analysis was used to detect the size of exosomes. **(C)** Exosomal protein levels of TSG101, CD9 and CD63 were detected by western blot. **(D)** qPCR validation of exosomal miRNAs levels. **(E)** The concentration of exosomes was detected by the BCA assay. **(F)** Dil-labeled exosomes were used to coculture with iBMDMs to visualize the internalization MAT-Exos by macrophage. **(G)** The concentrations of pro-inflammatory and anti-inflammatory cytokines in culture supernatants of iBMDMs. The mRNA expression levels **(H)**, the protein expression levels **(I)** and flow cytometry **(J)** were detected in iBMDMs in different groups. Data are expressed as means ± SD. *P < 0.05; **P < 0.01; ***P < 0.001; ****P < 0.0001 and n = 3 biological replicates.

Following our insights from the patients, we extended our investigation on a DNBS-induced mouse model of CD. We successfully isolated exosomes from MAT using established protocols, termed as mHt-exos groups and mN-exos groups, respectively. Comparative analyses using TEM imaging showed similar cup-shaped morphology (**Figure 2A**), while NTA demonstrated comparable size distributions (30–200 nm) between both exosome populations (**Figure 2B**). Western blot analysis revealed significantly elevated levels of exosomal markers (CD9, CD63, and TSG101) in mHt-exos (**Figures 2C, D**). An increased exosomal protein yield was observed when adipocytes were normalized (**Figure 2E**). Confocal microscopy confirmed effective internalization of Dil-labeled exosomes (30 μg/mL) by iBMDMs within 12 h (**Figure 2F**). mHt-exos treatment significantly altered the cytokine profile, increasing pro-inflammatory mediators (TNF-α, IL-6, MPO) while decreasing anti-inflammatory IL-10 in culture supernatants (**Figure 2G**). At the transcriptional level, mHt-exos upregulated M1-associated genes (CD86, iNOS and TNF-α) and downregulated M2 markers (Arg1 and CD206) (**Figure 2H**). Consistent with these findings, western blotting showed elevated M1 protein expression (**Figure 2I**), while flow cytometry confirmed an increase in CD86^+^ M1 macrophages (**Figure 2J**). Taken together, these findings demonstrate that mHt-exos preferentially drive macrophage polarization toward a pro-inflammatory M1 phenotype, which was consistent with the results in CD.

### mHt-exos aggravated ileocolonic anastomosis inflammation and promoted macrophage M1 polarization in a surgical model of IL10-knockout mice

Prior to performing animal experiments, we determined the amount of exos injected intraperitoneally by in our DSS-induced colitis model (**Supplementary Figure S1A**). In the vivo experiments, mice were treated with increasing doses of exosomes: 0 μg [saline], 50, 100, 200, 400 and 800 μg. It was observed that inflammatory alterations began to manifest in the mice following administration of 200 μg: Colon length tended to be shorter, and histological inflammation tended to worsen with an increase in the Ht-Exos dose (**Supplementary Figures S1B–E**). With an increase in dose, the degree of intestinal inflammation became increasingly serious, as reflected by the histopathological inflammatory score (**Supplementary Figures S1F, G**).

**Figure 3:**
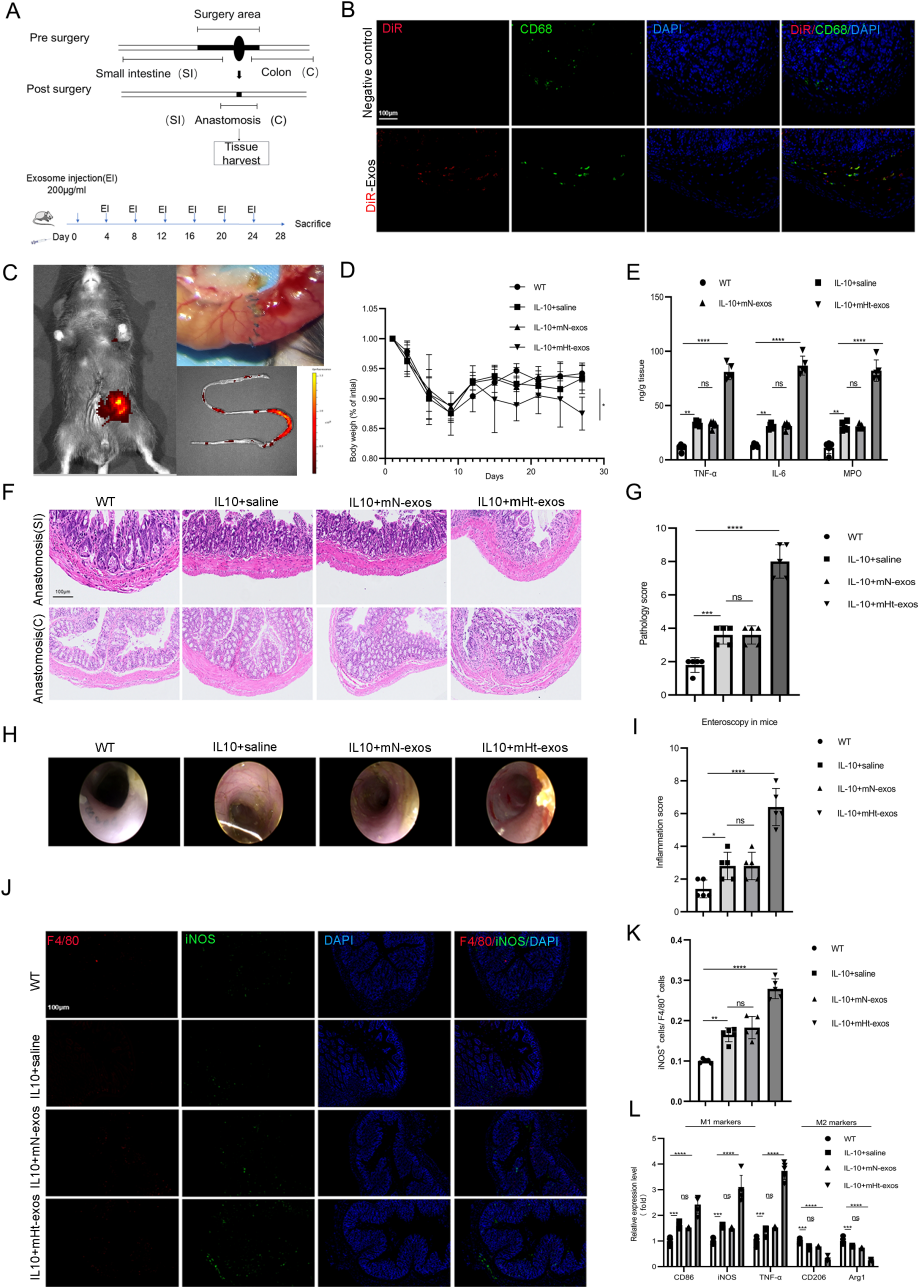
mHt-exos aggravated ileocolonic anastomosis inflammation and promoted macrophage M1 polarization. **(A)** Schematic diagram of ileocecal resection (ICR) and tissue harvest. **(B)** DiR-labeled -Exos were intraperitoneal (IP) injected into the mice and the tissues were then stained with FITC-labeled CD86. **(C)** Ileocolonic anastomosis exhibition. DiR dye was used to mark the exosomes and the DiR-labelled exosomes were intraperitoneal injected into the mice and tracked ex vivo via an imaging system. **(D)** Relative body weight in different groups. **(E)** The concentrations of pro-inflammatory and anti-inflammatory cytokines (TNF-α, IL-6, MPO) in the different groups. The different groups were assessed through H&E **(F)** DAI score **(G)**, Colonoscopy **(H, I)** respectively. **(J, K)** Double immunofluorescent staining was conducted to further detect the polarization of macrophages in different groups. **(L)** mRNA expression levels of CD86, iNOS, TNF-α, CD206, Arg1 and IL-10 were detected by qRT-PCR. Data are expressed as means ± SD. *P < 0.05; **P < 0.01; ***P < 0.001; ****P < 0.0001 and n = 3 biological replicates.

In order to better simulate the situation of anastomotic recurrence in clinical patients, we used an IL-10^-/-^ mouse model of ileocecal resection (**Figure 3A**). DiR-labeled exosomes successfully localized to anastomotic sites within 12 h, which verified whether exosomes are able to act on the anastomotic stoma (**Figures 3B, C**). In our study, mice were categorized into four groups: WT Group and IL-10+saline Group, IL-10+mN-exos Group, and IL-10+mHt-exos Group. Inflammation assessment was performed at 21 postoperative days. The results revealed that compared with controls, mHt-exos-treated mice exhibited significant weight loss (**Figure 3D**), anastomosis inflammation (**Figure 3E**), more severe endoscopic inflammation and higher histopathological inflammatory scores (**Figures 3F–I**). Molecular analyses showed that Ht-exos promoted M1 macrophage polarization. Specifically, mHt-exos elevated iNOS, TNF-α, and IL-6 expression; reduced Arg1, CD206; and increased iNOS^+^ macrophages (**Figures 3J–L**). The comprehensive data from our mouse model experiments demonstrate that mHt-exos exacerbates anastomosis inflammation by enhancing M1 macrophage polarization.

### An enrichment of exosomal miR-26b-3p

**Figure 4:**
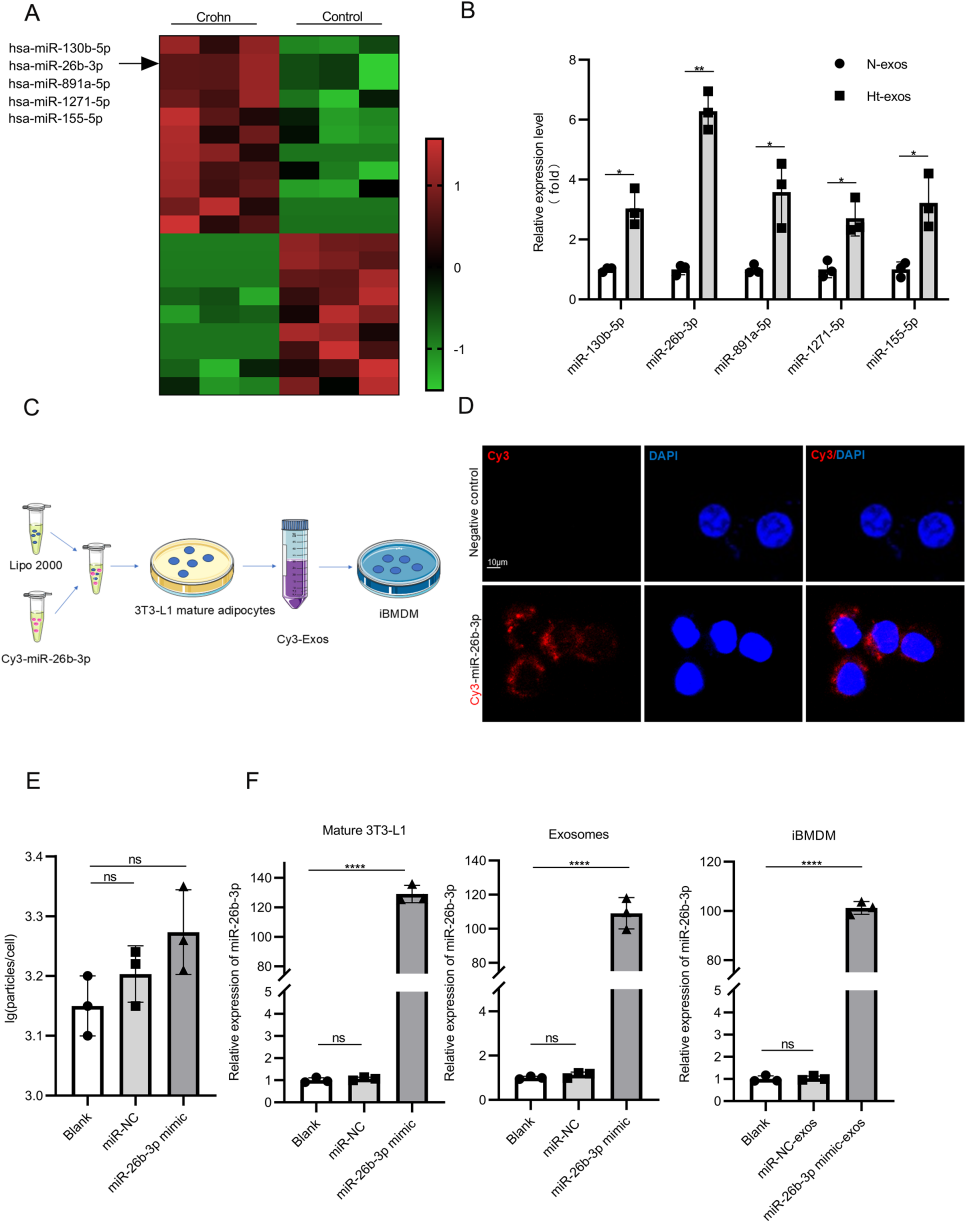
An enrichment of exosomal miR-26b-3p. **(A)** Heat map exhibiting 11 upregulated and 9 downregulated miRNAs [fold-change ≥1.5, p < 0.05]. **(B)** The expression levels of five potential targets were detected by qRT-PCR. **(C)** 3T3-L1 mature adipocytes were transfected with Cy3-labelled miR-26b-3p mimics and the corresponding exosomes were isolated. The iBMDMs were then treated with these exosomes. **(D)** In order to observe the exosomal miR-26b-3p transfer from 3T3-L1 mature adipocytes to macrophages, 3T3-L1 mature adipocytes were transfected with miR-26b-3p mimics that were labeled with Cy3, and the iBMDMs were treated with the appropriate exosomes. Cy3 immunofluorescence intensity was then used to confirmed the shuttling of exosomal Cy3- miR-26b-3p into recipient macrophages. **(E)** Number of exosomes from equal volumes of culture medium obtained from differentiated 3T3-L1 adipocytes transfected with miR-NC mimic or miR-26b-3p mimic. **(F)** The mRNA expression levels of miR-130b-5p levels in 3T3-L1 mature adipocytes, exosomes and iBMDMs. Data are expressed as means ± SD. *P < 0.05; **P < 0.01; ***P < 0.001; ****P < 0.0001 and n = 3 biological replicates.

To explore the molecular mechanisms, microarray analysis derived from Crohn groups and Control groups was performed. The heat map showed revealed 19 differentially expressed miRNAs (11 upregulated and 8 downregulated; p<0.05, FC>1.5) (**Figure 4A**). We particularly focused on miR-26b-3p owing to its known pro-inflammatory roles (29, 30). At the same time, we found miR-26b-3p had a higher level in mHt-exos compared with mN-exos (**Figure 4B**). In order to see if transfection of miR-26b-3p would have any effect on adipocytes, the following experiments were performed. Through fluorescent tracing experiments in which 3T3-L1 adipocytes were transfected with Cy3-labeled miR-26b-3p mimics (**Figures 4C, D**), we found that exosome production was not affected (**Figure 4E**) and miR-26b-3p successful transfer to recipient iBMDMs, while miR-26b-3p enrichment in donor adipocytes, secreted exosomes, and recipient macrophages (**Figure 4F**). These findings demonstrate a functional exosomal shuttle mechanism for adipocyte-macrophage communication that may drive inflammation activation.

### mHt-exos facilitated macrophage M1 polarization through exosomal miR-26b-3p *in vitro* and *in vivo*

**Figure 5:**
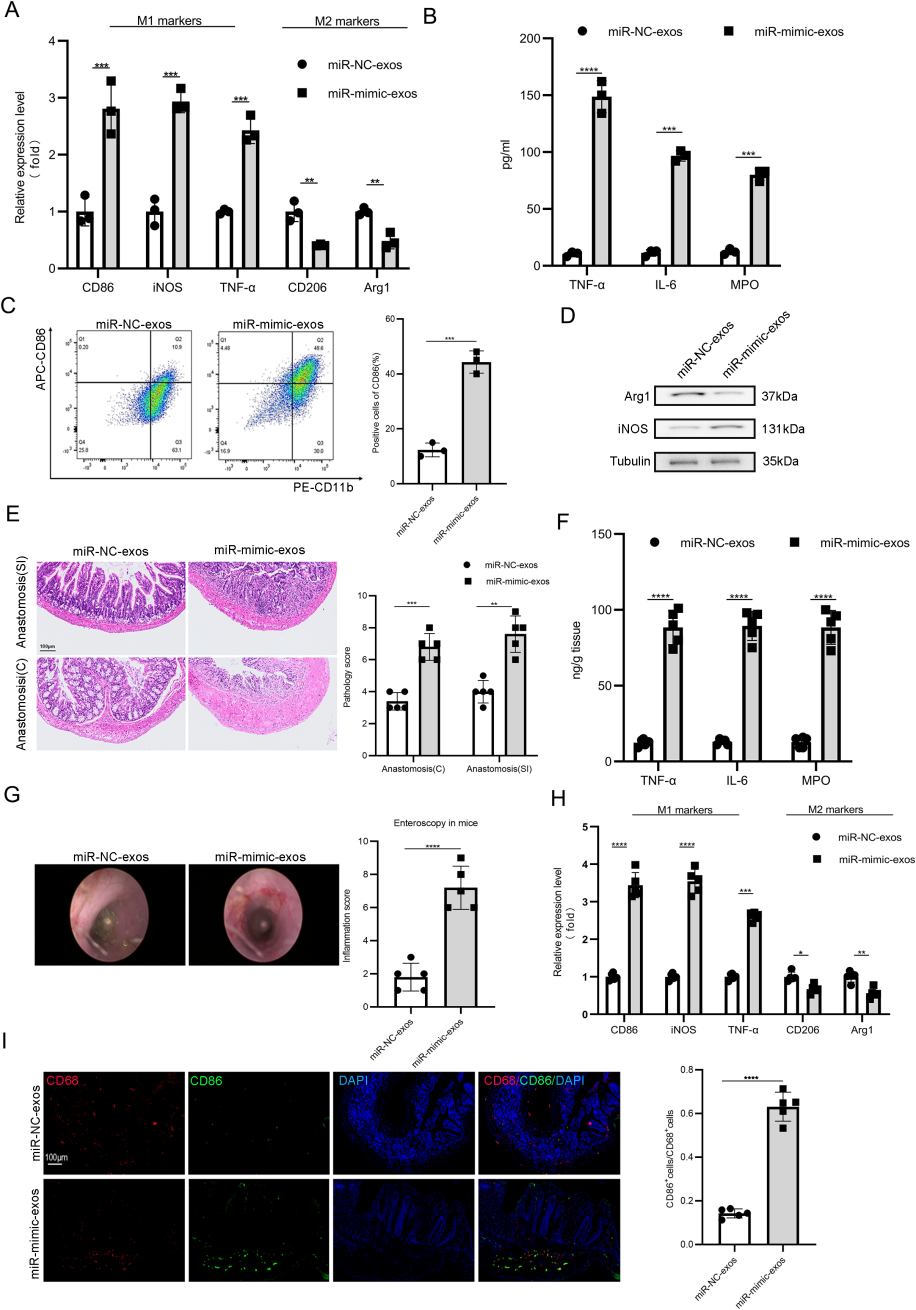
mHt-exos facilitated macrophage M1 polarization through exosomal miR-26b-3p *in vitro* and *in vivo*. The mRNA expression levels **(A)**, the concentrations of pro-inflammatory and anti-inflammatory cytokines **(B)**, flow cytometry **(C)** and the protein expression levels **(D)** were detected in iBMDMs in different groups. The different groups were assessed through H&E **(E)**, The concentrations of pro-inflammatory and anti-inflammatory cytokines **(F)**, Colonoscopy **(G)** and the mRNA expression levels **(H)**, respectively. **(I)** Double immunofluorescent staining was conducted to further detect the polarization of macrophages in different groups. Data are expressed as means ± SD. *P < 0.05; **P < 0.01; ***P < 0.001; ****P < 0.0001, n = 5 mice in each group and n = 3 biological replicates.

To further validate the biochemical impact of these findings, the following experiments were performed. *In vitro*, iBMDMs co-cultured with exosomes from miR-26b-3p-overexpressing adipocytes (miR-mimic-exos) exhibited significantly elevated pro-inflammatory cytokines (TNF-α, IL-1β, IL-6) and reduced anti-inflammatory IL-10 compared to miR-NC-exos-treated controls (**Figures 5A, B**). Consistent with these findings, flow cytometry and western blotting confirmed enhanced M1 polarization in miR-mimic-exos group (**Figures 5C, D**). Moreover, *in vivo*, miR-mimic-exos intensified anastomotic inflammation, as evidenced by worsened histological scores, elevated endoscopic inflammation, increased pro-inflammatory cytokine levels, and decreased anti-inflammatory cytokine levels (**Figures 5E–G**). qRT-PCR and immunofluorescence analyses further demonstrated that miR-mimic-exos induced macrophage polarization toward the M1 phenotype (**Figures 5H, I**). The comprehensive data established mHt-exos promote M1 macrophage polarization through exosomal miR-26b-3p both *in vitro* and *in vivo*, highlighting exosomal miR-26b-3p was a key regulator of inflammatory responses across anastomosis inflammation.

### Mechanistic insights into miR-26b-3p/TRIM33/MAPK regulation of macrophage polarization

**Figure 6:**
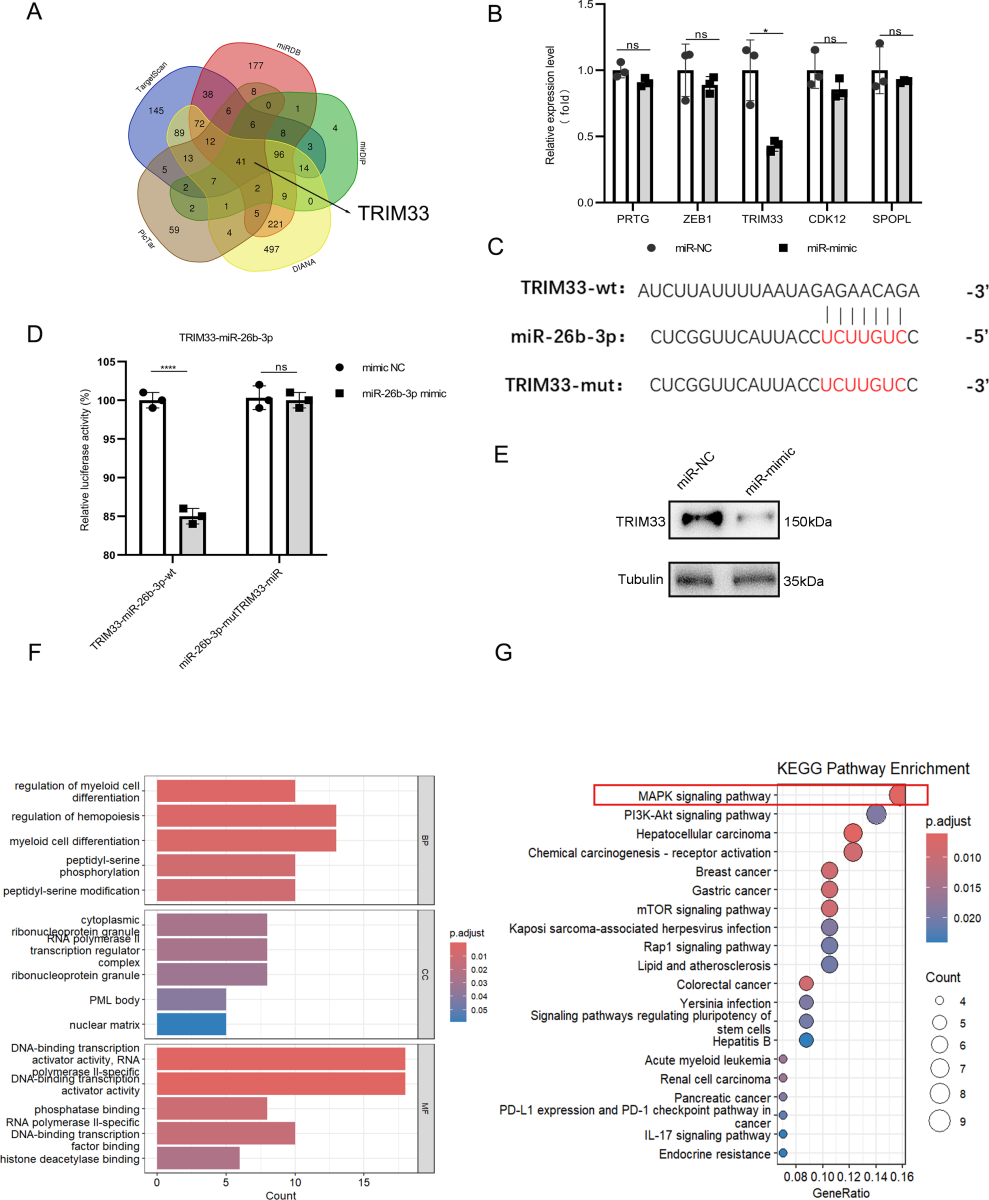
Exosomal miR-26b-3p targets TRIM33. **(A)** Five bioinformatics databases [TargetScan, miRDB, mirDIP, PicTar, and DIANA] were used to predict the downstream gene of miR-26b-3p. **(B)** The mRNA expression levels of five candidate molecules (PRTG, ZEB1, TRIM33, CDK12, and SPOPL). **(C, D)** Illustration of the complementary sequences between miR-26b-3p and TRIM33 3’UTR and relative luciferase activity assayed by the ratio of firefly-renilla luciferase activity following transfection with miR-26b-3p mimic compared with transfection with mimic NC. **(E)** Western blot analysis miR-26b-3p mimic compared with transfection with mimic NC. **(F, G)** Gene Ontology (GO) analysis and KEGG pathway enrichment analysis about miR-26b-3p. Data are expressed as means ± SD. *P < 0.05; ****P < 0.0001, n = 5 mice in each group and n = 3 biological replicates.

To further investigate the molecular basis of these changes, Bioinformatics analysis was performed. Using five prediction databases (TargetScan, miRDB, mirDIP, PicTar, and DIANA), TRIM33 was the primary target of miR-26b-3p (**Figures 6A, B**). This interaction was functionally validated through a (1) dual-luciferase assay revealing miR-26b-3p-mediated suppression of WT (but not mutant) TRIM33 reporter activity (**Figures 6C, D**) and (2) western blot confirmation of TRIM33 protein downregulation in miR-26b-3p-transfected iBMDMs (**Figure 6E**). Pathway analyses revealed the involvement of miR-26b-3p in protein translation (Gene Ontology) and inflammatory pathways, especially the MAPK signaling pathway (Kyoto Encyclopedia of Genes and Genomes) (**Figures 6F, G**).

To confirm whether miR-26b-3p played a crucial role in the exosomal-mediated M1 polarization of macrophages. We then transducted mHt with lentiviral vectors to knockdown (miR^KD^) miR-26b-3p, as well as the corresponding empty lentiviral vectors (miR-NC^KD^). Quantitative reverse transcription-polymerase chain reaction was used to identify the transduction efficiency (**Supplementary Figure S2A**). Exosomes were extracted from miR-NC^KD^-mHt and miR^KD^-mHt. The miR-26b-3p in miR^KD^-mHt was significantly reduced compared with the corresponding negative control in exos (**Supplementary Figure S2B**). A dramatic decrease was observed after coculture with the miR^KD^-mHt-exos in iBMDMs(**Supplementary Figure S2C**). miR^KD^-mHt-exos treatment significantly altered the cytokine profile, increasing pro-inflammatory mediators (TNF-α, IL-6, MPO) (**Supplementary Figure S2D**). At the transcriptional level, miR^KD^-mHt-exos upregulated M1-associated genes (CD86, iNOS and TNF-α) and downregulated M2 markers (Arg1 and CD206) (**Supplementary Figure S2E**). Consistent with these findings, western blotting showed elevated M1 protein expression (**Supplementary Figure S2H**), while flow cytometry confirmed an increase in CD86^+^ M1 macrophages (**Supplementary Figures S2F, G**).

**Figure 7:**
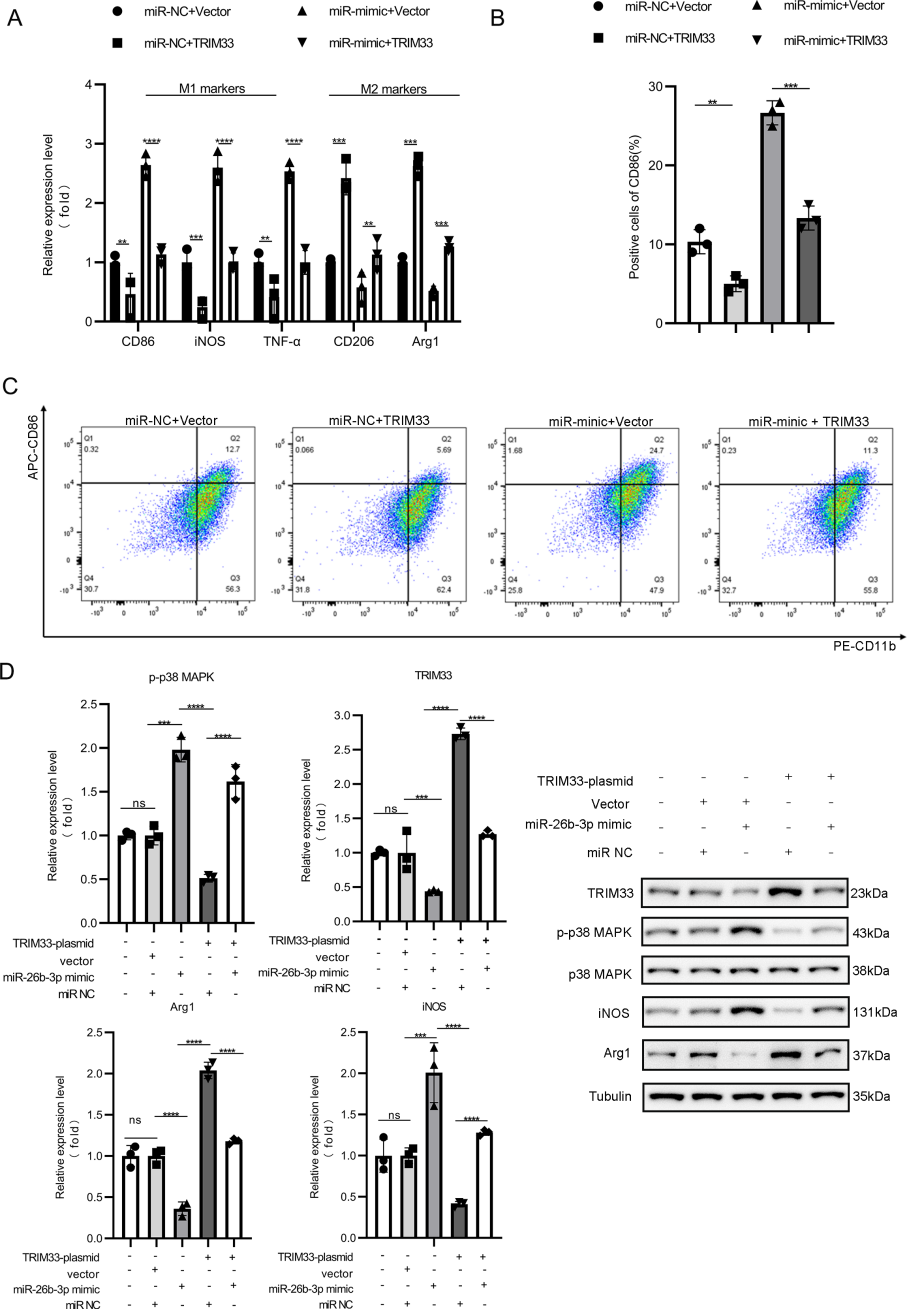
Exosomal miR-26b-3p modulates TRIM33 via MAPK signaling pathway to influence macrophage activation. The mRNA expression levels **(A)**, the positive cells of CD86 **(B)**, flow cytometry **(C)** and the protein expression levels **(D)** were detected in iBMDMs in different groups. Data are expressed as means ± SD. **P < 0.01; ***P < 0.001; ****P < 0.0001, n = 5 mice in each group and n = 3 biological replicates.

Functional studies demonstrated that miR-26b-3p mimic treatment activated the MAPK pathway (elevated p-p38MAPK) and M1 markers (iNOS, TNF-α), while TRIM33 overexpression exerted opposite effects, with these effects being reversed by miR-26b-3p co-transfection (**Figures 7A–D**). These results established a definitive TRIM33/MAPK axis through which exosomal miR-26b-3p drives macrophage polarization.

### Correlation between miR-26b-3p expression in MAT and ileocolonic anastomosis inflammation in patients with CD

**Figure 8:**
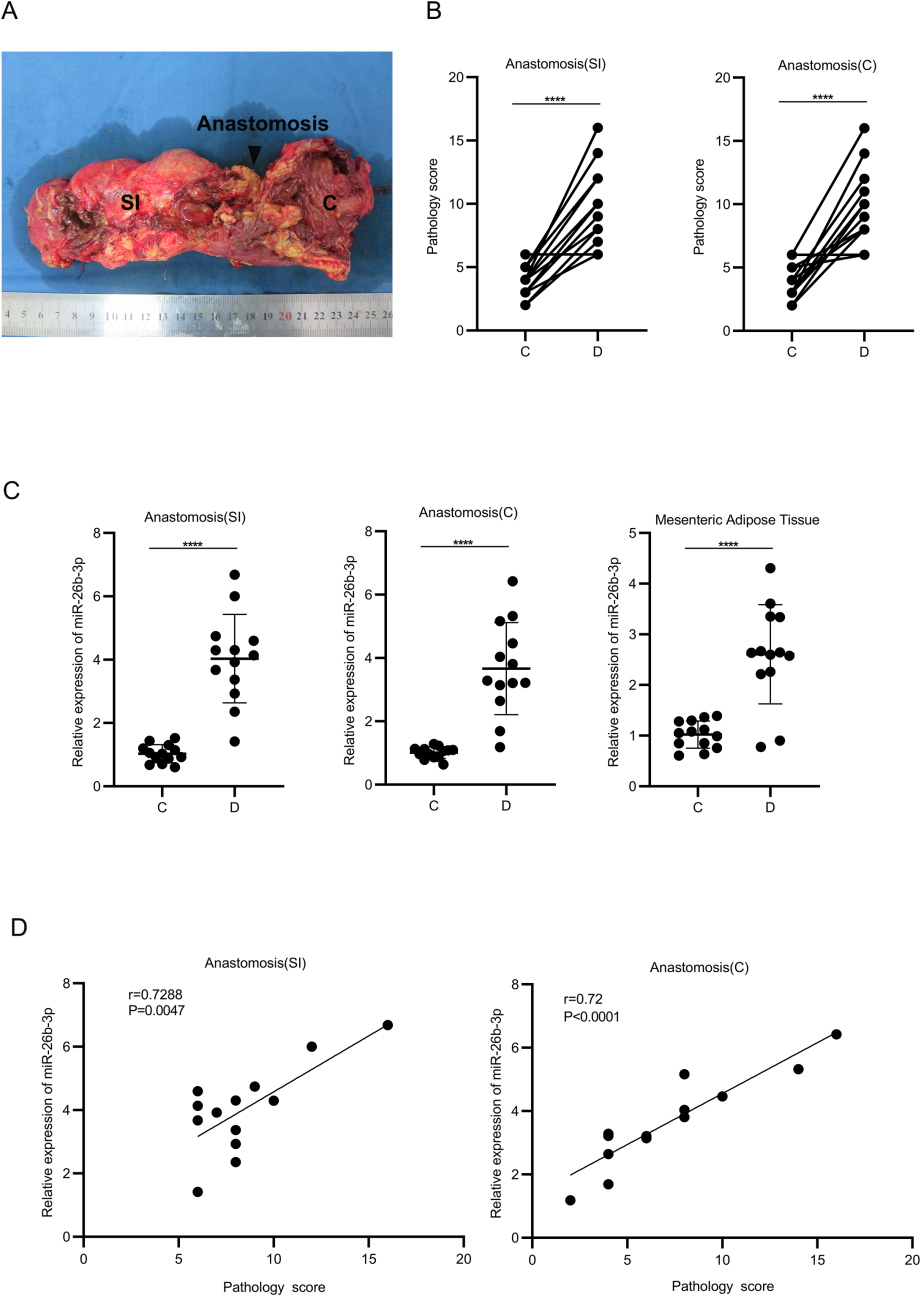
Correlation between miR-26b-3p expression, mesenteric adipose tissue, and ileocolonic anastomosis inflammation in Crohn’s disease patients. **(A)** Typical appearance of specimens. **(B)** The degree of the pathology score was clearly increased in the diseased intestine group. **(C)** The expression of miR-26b-3p in the ileocolonic anastomotic and the mesenteric fat tissue was then quantified by qRT-PCR. **(D)** The expression of miR-26b-3p in the diseased ileocolonic anastomotic was positively associated with the degree of the pathology score. ****P < 0.0001.

In order to observe whether it has potential clinical value, a number of clinical samples (**Figure 8A**) were selected for validation. Analysis of anastomotic specimens patients with and without CD revealed significantly elevated miR-26b-3p expression in both inflamed intestinal tissue and adjacent MAT in the CD group than in the control group (**Figures 8B, C**). Pathological scoring demonstrated a strong positive correlation between miR-26b-3p levels and inflammatory severity at anastomotic sites (**Figure 8D**). These findings suggest that miR-26b-3p could be a biomarker for postoperative inflammation progression as well as a therapeutic target for modulating M1 macrophage-driven inflammatory responses in CD.

## Discussion

Our findings suggest that residual diseased mesenteric fat exacerbates postoperative anastomotic inflammation through exosome-mediated mechanisms, which may contribute to inflammation recurrence at the surgical site. Mechanistically, exosomal miR-26b-3p targeted MAPK, which promoted intestinal inflammation and drove macrophage polarization toward the M1 phenotype via the MAPK signaling pathway.

Adipose tissue is considered a complex endocrine organ capable of producing cytokines, adipokines, and growth factors (14). Dysregulated adipose tissue secretes various exocrine factors that contribute to disease progression. Specifically, pathogenic adipose tissue may be a significant risk factor for atherosclerotic coronary heart disease, type 2 diabetes mellitus (T2DM), hypertension, and dyslipidemia (31). The major secretory product from adipose tissue is free fatty acids. A sustained and excessive net increase in circulating free fatty acids contributes to metabolic disease (32, 33). Similarly, patients with CD have demonstrated a disordered structure and functional dysregulation of MAT (3), which has been associated with disease progression, including intestinal fibrosis (34). Furthermore, mesenteric fat has been associated with lymphatic dysfunction (35). Exosomes derived from mesenteric adipose stem cells have been reported to alleviate intestinal inflammation (36). Contrastingly, we observed that MAT may exacerbate CD via exosome-mediated mechanisms.

Macrophages are highly plastic immune cells whose phenotypes and functions are dynamically regulated by various environmental cues (37–39). Broadly, activated macrophages can be categorized into pro-inflammatory M1-like and anti-inflammatory M2-like macrophages, which each have distinct metabolic profiles (40). For example, lipopolysaccharide-activated macrophages exhibit a glycolytic shift and are classified as M1 macrophages, while IL-4-stimulated macrophages adopt an anti-inflammatory M2 phenotype (41, 42). This plasticity allows macrophages to adapt their functional properties to local microenvironmental signals. Notably, exosomes derived from mesenchymal stem cells alleviated experimental colitis by promoting an M2-like phenotype (36). Moreover, administering stem cell-derived exosomes to mesenteric fat can ameliorate inflammatory changes (43). In contrast, we observed a novel mechanism whereby MAT-derived exosomes drove macrophage polarization toward the M1 phenotype both *in vitro* and *in vivo*, which resulted in elevated levels of pro-inflammatory cytokines. These findings highlight the dual role of exosomes in macrophage regulation; further, they suggest that mesenteric fat is a potential therapeutic target for intestinal inflammation by modulating macrophage activity.

Exosomes carry diverse cargo, including mRNA, miRNA, DNA, and cytokines (13). MicroRNAs are crucially involved in exosome-mediated paracrine communication given their ability to regulate gene expression. Further, miR-26b-3p has been implicated in various pathological processes. For example, it may accelerate the metastasis of hepatocellular carcinoma via the miR-26b-3p/MDM4 axis (44). In the etiology of idiopathic short stature (ISS), miR-26b-3p overexpression in ISS plasma exosomes leads to disorders in proliferation and endochondral ossification of growth plate cartilage via inhibition of AKAP2/ERK1/2 axis (45). However, its role in intestinal inflammation, particularly in CD, remains unclear.

We observed elevated levels of miR-26b-3p in Ht-exos. Specifically, these exosomes transferred miR-26b-3p to macrophages, which drove macrophage polarization toward the pro-inflammatory M1 phenotype and contributed to intestinal inflammation. Mechanistically, miR-26b-3p directly targets the 3'-UTR of TRIM33, which leads to its downregulation. TRIM33, which is an established regulator of inflammation, tumor immunity, and immune homeostasis, is a critical checkpoint in the inflammatory response. Furthermore, miR-26b-3p activated the p38-MAPK signaling pathway, a key regulator of inflammation and cancer. Although miR-26b-3p has been primarily linked to NF-dB pathway activation, our findings highlight its role in the MAPK pathway, providing new insights into its inflammatory mechanisms.

Using bioinformatic analysis, we predicted and experimentally validated a novel target of miR-26b-3p, which further expanded its functional repertoire. Notably, miR-26b-3p was significantly upregulated at anastomotic recurrence sites in patients with CD, in both the intestinal tissue and adjacent mesenteric fat. Notably, miR-26b-3p expression was positively correlated with inflammation scores and MAT involvement, which suggests that it may be a biomarker for monitoring and predicting anastomotic inflammation recurrence in CD. These findings underscore the clinical relevance of miR-26b-3p and highlight its potential as a therapeutic target in CD. A direct clinical application could be developing miR-26b-3p as a circulating biomarker. We hypothesize that exosomal miR-26b-3p might be detectable in the blood or even feces of patients. Serial monitoring of its levels in the perioperative and postoperative period could serve as an early warning signal for subclinical inflammation or impending anastomotic complication, preceding clinical symptoms or conventional markers like C-reactive protein. Preoperative assessment of miR-26b-3p levels in mesenteric tissue or serum might help identify patients at higher risk for aggressive postoperative disease course, enabling personalized surveillance strategies or early therapeutic intervention. Meanwhile, advanced biomaterial delivery systems can significantly enhance the therapeutic efficacy and clinical translation potential of exosome-based therapies (46–48). Combining mesenteric adipose tissue-derived exosomes (MAT-exos) or their key effector molecules (such as miR-26b-3p inhibitors) with smart biomaterials to construct a local sustained-release drug delivery system could significantly enhance the targeting and durability of treatment. This strategy is expected to greatly improve the therapeutic efficacy of exosome-based therapies and promote their clinical translation, potentially representing a next-generation local treatment approach for preventing postoperative recurrence in Crohn’s disease.

## Conclusion

Hypertrophic MAT in CD contributes to intestinal anastomotic inflammation, which is mainly derived by induction of macrophage polarization. Mechanistically, HtMAT-Exos promoted macrophage M1 polarization by targeting the p38-MAPK signaling pathway via exosomal miR-26b-3p.

## Data Availability

Raw data could not be uploaded to a public network disk because of patient privacy agreements and confidentiality requirements. The original contributions presented in the study are included in the article/**Supplementary Material**, further inquiries can be directed to the corresponding author.

## Supplementary material

Supplementary Figure 1 Dose-dependent protective effects of MAT-derived exosomes in DSS colitis. **(A)** Schematic diagram of exosomes were initially injected during the DSS administration. Colon length **(B, C)** and weight change **(D)** in each group. The concentrations of pro-inflammatory and anti-inflammatory cytokines in culture supernatants of iBMDMs The mRNA expression levels **(E)**. The different groups were assessed through DAI score **(G)** and H&E **(F)**. Data are expressed as means ± SD. *P < 0.05; **P < 0.01; ***P < 0.001; ****P < 0.0001 and n = 5 mice in each group.

Supplementary Figure 2 miR-26b-3p played a crucial role in the exosomal-mediated M1 polarization of macrophages. The miR-26b-3p expression levels were detected by qRT-PCR **(A–C)**. **(D)** The concentrations of pro-inflammatory and anti-inflammatory cytokines in culture supernatants of iBMDMs. The mRNA expression levels **(E)**, flow cytometry **(F, G)** and the protein expression levels **(H)** were detected in iBMDMs in different groups. Data are expressed as means ± SD. *P < 0.05; **P < 0.01; ***P < 0.001; ****P < 0.0001 and n = 3 biological replicates.
