# Supplementary figures and images for: The exosomal miR-26b-3p derived from Crohn’s disease-associated mesenteric adipose tissue induces M1 macrophage polarization and exacerbates ileocolonic anastomosis inflammation via the p38-MAPK signaling pathway
Source: Front Immunol. 2026 Feb 25;17:1754302. doi: 10.3389/fimmu.2026.1754302 (PMC12975433; doi:10.3389/fimmu.2026.1754302)

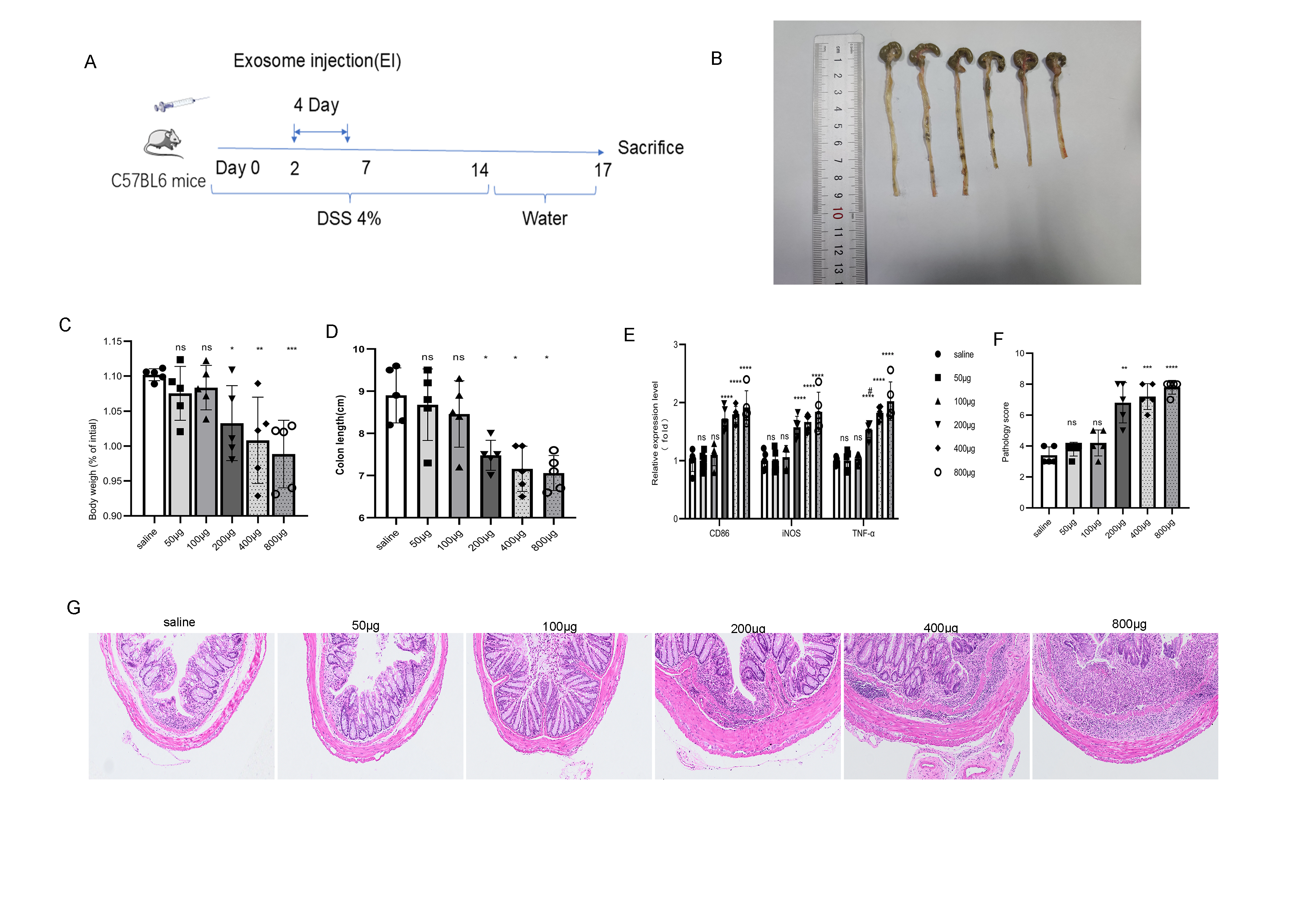

Supplement: Supplementary Figure 1 — Dose-dependent protective effects of MAT-derived exosomes in DSS colitis. (A) Schematic diagram of exosomes were initially injected during the DSS administration. Colon length (B, C) and weight change (D) in each group. The concentrations of pro-inflammatory and anti-inflammatory cytokines in culture supernatants of iBMDMs The mRNA expression levels (E). The different groups were assessed through DAI score (G) and H&E (F). Data are expressed as means ± SD. *P < 0.05; **P < 0.01; ***P < 0.001; ****P < 0.0001 and n = 5 mice in each group. [file Image1.tif]

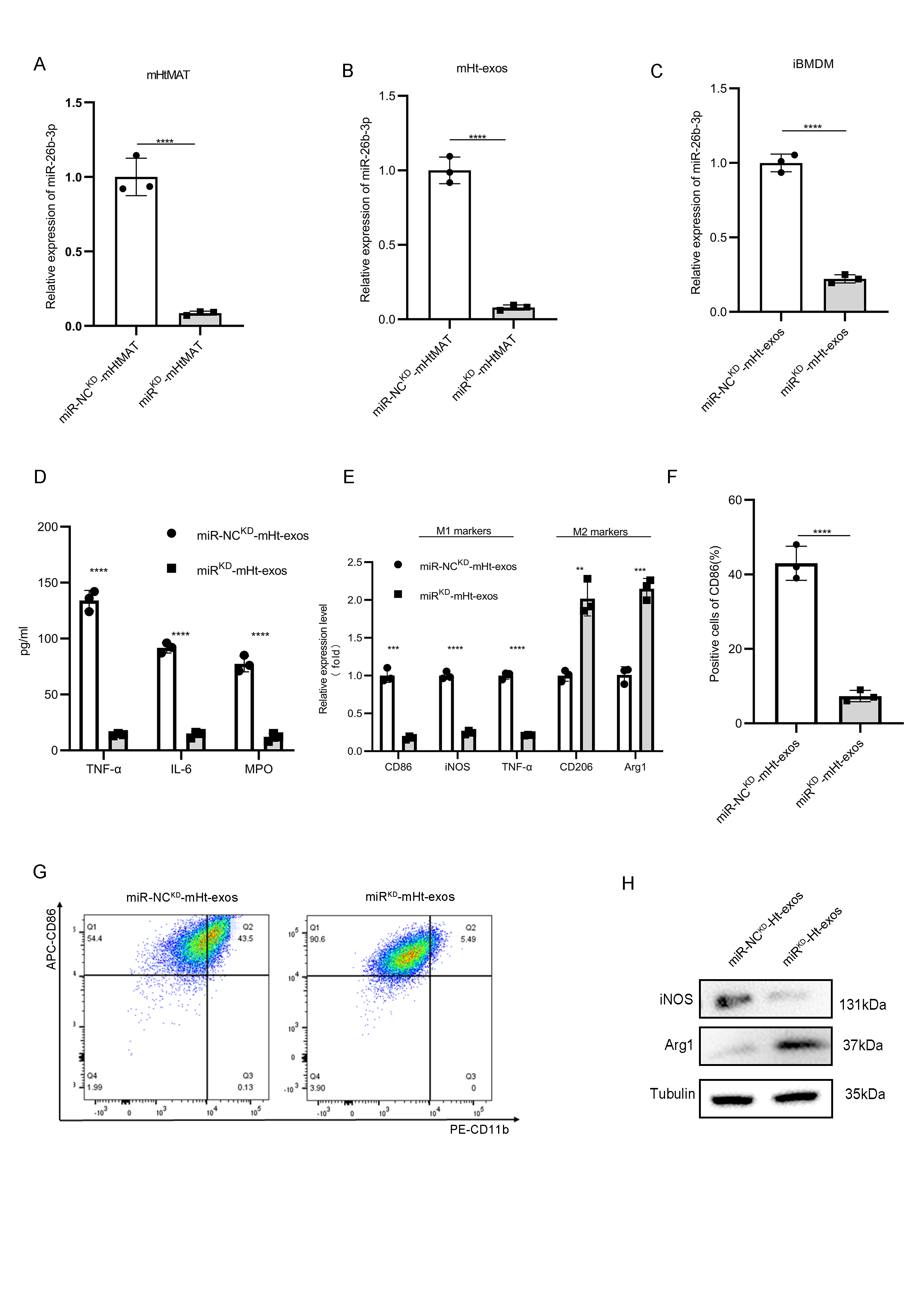

Supplement: Supplementary Figure 2 — miR-26b-3p played a crucial role in the exosomal-mediated M1 polarization of macrophages. The miR-26b-3p expression levels were detected by qRT-PCR (A–C). (D) The concentrations of pro-inflammatory and anti-inflammatory cytokines in culture supernatants of iBMDMs. The mRNA expression levels (E), flow cytometry (F, G) and the protein expression levels (H) were detected in iBMDMs in different groups. Data are expressed as means ± SD. *P < 0.05; **P < 0.01; ***P < 0.001; ****P < 0.0001 and n = 3 biological replicates. [file Image2.tif]
