# Supplementary material for: The exosomal miR-26b-3p derived from Crohn’s disease-associated mesenteric adipose tissue induces M1 macrophage polarization and exacerbates ileocolonic anastomosis inflammation via the p38-MAPK signaling pathway
Source: Front Immunol. 2026 Feb 25;17:1754302. doi: 10.3389/fimmu.2026.1754302 (PMC12975433; doi:10.3389/fimmu.2026.1754302)
Supplement: Supplementary file 3 [file Table1.docx]

| Table 1. Patients Characteristics. |  |
| --- | --- |
|  | CD (n=15) |
| Gender, male, n (%) | 11 (73.3) |
| BMI | 17.7±2.3 |
| Age | 33.4±8.7 |
| Duration disease (years), median (IQR) | 3.7(1.3-7.2) |
| Smoking, n (%) | 5(30) |
| Disease location, L3 (ileocolonic) | 15(100) |
| Disease behavior, B3 (stricturing) | 15(100) |
| Perianal disease history, n (%) | 4(22.2) |
| Preoperative medications, n (%) |  |
| 5-ASA | 9(60.0) |
| Immunosuppressor | 7(46.6) |
| Anti-TNF | 3(20.0) |
| Steroids | 5(33.3) |
| Preoperative parameters |  |
| CRP, median (IQR) | 8.7(1.65-8.2) |
| ALB, median (IQR) | 38.2(37.0-40.7) |

ALB: albumin (g/L); anti-TNF: anti-tumor necrosis factor; BMI: body mass index; CRP: C-reactive protein (mg/L); IQR: interquartile range; 5-ASA: 5-aminosalicylic acid.
