# Supplementary material for: The exosomal miR-26b-3p derived from Crohn’s disease-associated mesenteric adipose tissue induces M1 macrophage polarization and exacerbates ileocolonic anastomosis inflammation via the p38-MAPK signaling pathway
Source: Front Immunol. 2026 Feb 25;17:1754302. doi: 10.3389/fimmu.2026.1754302 (PMC12975433; doi:10.3389/fimmu.2026.1754302)
Supplement: Supplementary file 4 [file Table2.docx]

| **Table 2. Detailed information about the 15 patients used for exosomes isolation.** | | | | | | | | | | | | | | | |
| --- | --- | --- | --- | --- | --- | --- | --- | --- | --- | --- | --- | --- | --- | --- | --- |
| Patient_ID | Gender | Age | Duration disease (years) | BMI | Perianal disease history | Disease location | Disease behavior | Smoking | 5-ASA | Immunosuppressor | Anti-TNF | | Steroids | ALB | CRP |
| 1 | male | 47 | 10.2 | 17.76 | 0 | L3 | B3 | 0 | 0 | 0 | 1 | | 1 | 37.9 | 41.9 |
|  |  |  |  |  |  |  |  |  |  |  |  | |  |  |  |
| 2 | male | 20 | 3.4 | 16.44 | 0 | L3 | B3 | 0 | 1 | 0 | 0 | | 0 | 38.3 | 18.5 |
| 3 | female | 37 | 12.1 | 18.73 | 0 | L3 | B3 | 0 | 0 | 1 | 0 | | 0 | 38.1 | 5.8 |
| 4 | male | 30 | 3.7 | 15.57 | 1 | L3 | B3 | 0 | 1 | 1 | 0 | | 0 | 45.6 | 1.8 |
| 5 | male | 26 | 1.3 | 17.96 | 0 | L3 | B3 | 0 | 0 | 0 | 0 | | 0 | 40.2 | 2.7 |
| 6 | male | 36 | 3.8 | 16.79 | 0 | L3 | B3 | 0 | 1 | 0 | 0 | | 0 | 33.1 | 4.4 |
| 7 | female | 29 | 9.4 | 18.21 | 0 | L3 | B3 | 0 | 1 | 1 | 0 | | 0 | 36.2 | 9 |
| 8 | male | 36 | 10.4 | 15.92 | 0 | L3 | B3 | 0 | 0 | 0 | 1 | | 0 | 26.6 | 42.2 |
| 9 | male | 26 | 0.5 | 17.3 | 1 | L3 | B3 | 1 | 0 | 1 | 0 | | 0 | 41.9 | 1.3 |
| 10 | female | 41 | 5.2 | 18 | 0 | L3 | B3 | 0 | 1 | 0 | 0 | | 0 | 38.7 | 3.1 |
| 11 | male | 32 | 6.7 | 17.54 | 0 | L3 | B3 | 0 | 0 | 1 | 0 | | 0 | 42.2 | 0.5 |
| 12 | female | 23 | 6.5 | 16.44 | 0 | L3 | B3 | 0 | 1 | 0 | 0 | 0 | | 37 | 1.6 |
| 13 | female | 40 | 1.2 | 20.03 | 0 | L3 | B3 | 0 | 0 | 0 | 1 | 0 | | 39.4 | 1.1 |
| 14 | female | 45 | 3.2 | 19.72 | 0 | L3 | B3 | 0 | 1 | 1 | 0 | 0 | | 37.1 | 4.3 |
| 15 | female | 41 | 6.8 | 19.72 | 0 | L3 | B3 | 0 | 0 | 1 | 0 | 0 | | 32.7 | 9.6 |
