# Supplementary material for: The exosomal miR-26b-3p derived from Crohn’s disease-associated mesenteric adipose tissue induces M1 macrophage polarization and exacerbates ileocolonic anastomosis inflammation via the p38-MAPK signaling pathway
Source: Front Immunol. 2026 Feb 25;17:1754302. doi: 10.3389/fimmu.2026.1754302 (PMC12975433; doi:10.3389/fimmu.2026.1754302)
Supplement: Supplementary file 5 [file Table3.docx]

| **Table 3**. **Primer Sequences** | | |
| --- | --- | --- |
| TNF-α | Forward | 5'-GTT CTA TGG CCC AGA CCC TCA CA-3' |
|  | Reverse | 5'-TAC CAG GGT TTG AGC TCA GC-3' |
| IL-1β | Forward | 5'-GAA ATG CCA CCT TTT GAC AGT G-3' |
|  | Reverse | 5'-TGG ATG CTC TCA TCA GGA CAG-3' |
| IL-6 | Forward | 5'-GCC CAC CAA GAA CGA TAG TCA-3' |
|  | Reverse | 5'-CAA GAA GGC AAC TGG ATG GAA-3' |
| IL-10 | Forward | 5'-TCAAGGCGCATGTGAACTCC-3' |
|  | Reverse | 5'-GATGTCAAACTCACTCATGGCT-3' |
| iNOS | Forward | 5'-GCTCGCTTTGCCACGGACGA-3' |
|  | Reverse | 5'-AAGGCAGCGGGCACATGCAA-3' |
| CD86 | Forward | 5'-TCAATGGGACTGCATATCTGCC-3' |
|  | Reverse | 5'-GCCAAAATACTACCAGCTCACT-3' |
| CD206 | Forward | 5‘-CAAGGAAGGTTGGCATTT-3’ |
|  | Reverse | 5'-CCTTTCAGTCCTTTGCAAGC-3' |
| Arg1 | Forward | 5'-CTATGTGTCATTTGGGTGGA-3' |
|  | Reverse | 5'-TCTGGGAACTTTCCTTTCAG-3' |
| β-actin | Forward | 5'-GTG AAA AGA TGA CCC AGA TCA T-3' |
|  | Reverse | 5'-GCT TCT CTT TGA TGT CAC GCA CGA T-3' |
| mir-26b-3p | Forward | 5'-TGCGCCCTGTTCTCCATTACT-3' |
|  | Reverse | 5'-GACGGCGGCTAAGAAATATG-3' |
| Mapk11(m) | Forward | 5’-GCTGCTCGCCCTTTCCAATCG-3’ |
|  | Reverse | CGTGCTTCAGGTGCTTGAGTAGG |
| Mapk12 (m) | Forward | GTTCAGAAGCTACAGAGTGCAGAGG |
|  | Reverse | GCTTGCGTTGGTCAGGACAGAG |
| Mapk13 (m) | Forward | GGGATGGAATTCAGCGAGGATAAGG |
|  | Reverse | ACAGTCTTCATTCACAGCCAGGTTG |
| Mapk14 (m) | Forward | CTGGCTCGGCACACTGATGATG |
|  | Reverse | GCCCACGGACCAAATATCCACTG |
