# Supplementary material for: The exosomal miR-26b-3p derived from Crohn’s disease-associated mesenteric adipose tissue induces M1 macrophage polarization and exacerbates ileocolonic anastomosis inflammation via the p38-MAPK signaling pathway
Source: Front Immunol. 2026 Feb 25;17:1754302. doi: 10.3389/fimmu.2026.1754302 (PMC12975433; doi:10.3389/fimmu.2026.1754302)
Supplement: Supplementary file 6 [file Table4.docx]

| **Table 4**. Detail of the histological score of inflammation | | | | |
| --- | --- | --- | --- | --- |
| Scores  Parameters | 0 | 1 | 2 | 3 |
| Mucosal architecture change | None | Focal and mild | Mmultifocal or diffuse and mild to moderate | Multifocal or diffuse and severe |
| Mononuclear cell infiltration | Within normal limits | Slightly increased infiltrate in the lamina propria | Dense infiltrate in the lamina propria | Cell aggregates in the mucosa or submucosa |
| Neutrophil infiltration | None | In the lamina propria with or without cryptitis | One or more crypt abscesses | Infiltration of neutrophils in the mucosa or submucosa |
| Epithelial defects | None | Unequivocable focal erosion | Multifocal erosion | Ulceration |
| Goblet cell loss | None | Focal | Multifocal | Generalized |
