# Supplementary material for: The exosomal miR-26b-3p derived from Crohn’s disease-associated mesenteric adipose tissue induces M1 macrophage polarization and exacerbates ileocolonic anastomosis inflammation via the p38-MAPK signaling pathway
Source: Front Immunol. 2026 Feb 25;17:1754302. doi: 10.3389/fimmu.2026.1754302 (PMC12975433; doi:10.3389/fimmu.2026.1754302)
Supplement: Supplementary file 7 [file Table5.docx]

| Table 5. Patients Characteristics. | | | |
| --- | --- | --- | --- |
|  | CD (n=13) | non-CD (n=10) | p value |
| Gender, male, n (%) | 14(68.4) | 6(60) | 0.70 |
| BMI | 16.6±2.1 | 21.7±2.6 | <0.01 |
| Age | 28.6±7.5 | 53.1±15.2 | <0.01 |
| Duration disease (years) , median (IQR) | 3.0(2.0-7.0) | — | — |
| Disease location, n (%) |  |  |  |
| L1 (ileal) | 3(23.1) | — | — |
| L3 (ileocolonic) | 10(76.9) | — | — |
| Perianal disease history, n (%) | 4(30.79) | — | — |
| Disease behavior, B3 (stricturing), n (%) | 13(100) | — | — |
| Preoperative medications, n (%) |  |  |  |
| 5-ASA | 3(23.1) | — | — |
| Immunosuppressor | 1(7.6) | — | — |
| Anti-TNF | 2(15.3) | — | — |
| Steroids | 1(7.6) | — | — |
| Preoperative parameters |  |  |  |
| CRP, median (IQR) | 7.6(0.8-7.5) | 3.0(1.6-4.2) | 0.98 |
| ALB, median (IQR) | 41.1(37.2-43.7) | 41.8(38.5-47.0) | 0.51 |
| ALB: albumin (g/L); anti-TNF: anti-tumor necrosis factor; BMI: body mass index; CRP: C-reactive protein (mg/L); IQR: interquartile range; 5-ASA: 5-aminosalicylic acid. | | | |
|  | | | |
